# “Cell-Free Synthetic Biology”: Synthetic Biology Meets Cell-Free Protein Synthesis

**DOI:** 10.3390/mps2040080

**Published:** 2019-10-08

**Authors:** Seok Hoon Hong

**Affiliations:** Department of Chemical and Biological Engineering, Illinois Institute of Technology, Chicago, IL 60616, USA; shong26@iit.edu; Tel.: +1-312-567-8950

Since Nirenberg and Matthaei used cell-free protein synthesis (CFPS) to elucidate the genetic code in the early 1960s [1], the technology has been developed over the course of decades and applied to studying both fundamental and applied biology [2]. Cell-free synthetic biology integrating CFPS with synthetic biology has received attention as a powerful and rapid approach to characterize and engineer natural biological systems. The open nature of cell-free (or in vitro) biological platforms compared to in vivo systems brings an unprecedented level of control and freedom in design [3]. This versatile engineering toolkit has been used for debugging biological networks, constructing artificial cells, screening protein libraries, prototyping genetic circuits, developing biosensors, producing metabolites, and synthesizing complex proteins including antibodies, toxic proteins, membrane proteins, and novel proteins containing nonstandard (unnatural) amino acids. The *Methods and Protocols* “Cell-Free Synthetic Biology” Special Issue consists of a series of reviews, protocols, benchmarks, and research articles describing the current development and applications of cell-free synthetic biology in diverse areas.

Although interest in CFPS is growing, new users often face technical and functional issues in choosing and executing the CFPS platform that best suits their needs. An extensive review article by Gregorio et al. [4] provides a guide to help new users overcome the barriers to implementing CFPS platforms in research laboratories. CFPS platforms derived from diverse microorganisms and cell lines can be divided into two categories, including high adoption and low adoption platforms, by clarifying the similarities and differences among cell-free platforms. Various applications have been achieved by using each of these platforms. The authors also review methodological differences between platforms and the instrumental requirements for their preparation. New users can determine which type of cell-free platform could be used for their needs.

Another review article by Jeong et al. [5] summarizes the use of cell-free platforms for engineering synthetic biological circuits and systems. Because synthetic biological systems have become larger and more complex, deciphering the intricate interactions of synthetic systems and biological entities is a challenging task. Cell-free synthetic biology approaches can facilitate rapid prototyping of synthetic circuits and expedite the exploration of synthetic system designs beyond the confines of living organisms. Cell-free platforms can also provide a suitable platform for the development of DNA nanostructures, riboregulators, and artificial cells, and can enable validation of mathematical models for understanding biological regulation.

Incorporating nonstandard amino acids into proteins is an important technology to improve the understanding of biological systems as well as to create novel proteins with new chemical properties, structures, and functions. Improvements in CFPS systems have paved the way to accurate and efficient incorporation of nonstandard amino acids into proteins [6]. Gao et al. [7] describe a rapid and simple method to synthesize unnatural proteins in a CFPS system based on *Escherichia coli* crude extract by using an unnatural orthogonal translational machinery. This protocol provides a detailed procedure for using a CFPS system to synthesize unnatural proteins on demand. 

In CFPS systems, the activity of the crude extract is crucial to ensure high-yield protein synthesis and to minimize batch-to-batch variations in the cell-free reaction. Kim et al. [8] describe a practical method for the preparation and optimization of crude extract from genomically engineered *E. coli* strains [9]. This protocol summarizes entire steps of CFPS from cell growth to harvest, from cell lysis to dialysis, and from cell-free reaction setup to protein quantification. Of note, this method can be easily applied to other commercially available or laboratory stock *E. coli* strains to produce highly active crude extracts.

Because CFPS does not use living cells, toxic proteins can be produced in CFPS at high yield. Jin et al. [10] report that colicins, antimicrobial toxins, can be synthesized and optimized through CFPS at high-yield and activity. Chaperone-enriched *E. coli* extracts significantly enhance the protein solubility. Further modification of the system, such as by including the immunity protein that binds to the colicin, improves the cytotoxic activity of colicin. This study demonstrates that CFPS is a viable platform for optimal production of toxic proteins. 

Another optimization of CFPS systems by Yang et al. [11] is applied to produce biosimilar therapeutics. Posttranslational modification of mammalian proteins in prokaryotic systems is challenging. However, producing an active form of tissue plasminogen activator containing 17 disulfide bonds can be achieved in an *E. coli*-based CFPS by overexpressing or supplementing with disulfide bond isomerase and optimizing the buffer conditions during the reaction. This study represents an important step toward the development of *E. coli*-based CFPS technology for rapid, inexpensive, on-demand production of biotherapeutics. 

Eukaryotic CFPS systems can serve as alternative production systems for mammalian proteins that exhibit insufficient protein folding or posttranslational modification in prokaryotic CFPS systems. Thoring et al. [12] demonstrate that eukaryotic cell-free systems based on eukaryotic lysates have the potential to produce druggable protein targets. WNT proteins and the cytosolically produced hTERT enzyme have been produced and optimized in eukaryotic cell-free systems. The improvement of eukaryotic CFPS platforms has the potential to accelerate drug development pipelines. 

In addition to protein production, cell-free systems provide great benefits in advancing metabolic engineering. Lim and Kim [13] review recent developments and prospects of cell-free metabolic engineering, which, in comparison to cell-based metabolic processes, has the benefits of operational simplicity, high conversion yield and productivity, and no environmental release of engineered microorganisms. This article summarizes the importance of configuring cell-free enzyme synthesis and establishing cell-free metabolic engineering in the development of directly programmable metabolic engineering platforms. 

I believe that the collection of articles in the “Cell-Free Synthetic Biology” Special Issue of *Methods and Protocols* will provide researchers with both a comprehensive understanding of diverse aspects of cell-free synthetic biology and practical methods to apply cell-free synthetic biology tools and knowledge to advance their studies.
